# Melatonin suppresses autophagy in type 2 diabetic osteoporosis

**DOI:** 10.18632/oncotarget.10538

**Published:** 2016-07-11

**Authors:** Wei-Lin Zhang, Hong-Zheng Meng, Rui-Fei Yang, Mao-Wei Yang, Guang-Hong Sun, Jun-Hua Liu, Peng-Xu Shi, Fei Liu, Bo Yang

**Affiliations:** ^1^ Department of Orthopedics, the First Hospital of China Medical University, Shenyang, Liaoning, China; ^2^ School of Medical Applied Technology, Shenyang Medical College, Shenyang, Liaoning, China

**Keywords:** melatonin, type 2 diabetes osteoporosis, osteoblast, autophagy, ERK

## Abstract

Type 2 diabetes mellitus is often complicated by osteoporosis, a process which may involve osteoblast autophagy. As melatonin suppresses autophagy under certain conditions, we its investigated the effects on bone autophagy during diabetes. We first assessed different body parameters in a diabetic rat model treated with various concentrations of melatonin. Dynamic biomechanicalmeasurements, bone organization hard slice dyeing and micro-CT were used to observe the rat bone microstructure, and immunohistochemistry was used to determine levels of autophagy biomarkers. We also performed in vitro experiments on human fetal osteoblastic (hFOB1.19) cells cultured with high glucose, different concentrations of melatonin, and ERK pathway inhibitors. And we used Western blotting and immunofluorescence to measure the extent of osteogenesis and autophagy. We found that melatonin improved the bone microstructure in our rat diabetes model and reduced the level of autophagy(50 mg/kg was better than 100 mg/kg). Melatonin also enhanced osteogenesis and suppressed autophagy in osteoblasts cultured at high glucose levels (10 μM was better than 1 mM). This suggests melatonin may reduce the level of autophagy in osteoblasts and delay diabetes-induced osteoporosis by inhibiting the ERK signaling pathway.

## INTRODUCTION

Due to economic development, the incidence of chronic diseases such as diabetes is rising every year. China has the world's largest population of diabetics, and the age-standardized incidence of diabetics was 9.6 and 9.2 per 1000 person-years in men and women, respectively [1]. Various complications of diabetes significantly impair human health. In one survey, the risk of fractures was found to be much higher in patients with diabetes than in those without it [2]. Complications related to fractures reduce the quality of life and health of diabetic patients, cause a heavy economic burden, and have other deleterious effects on society. As the incidence of type 2 diabetes is far greater than that of type 1 diabetes, there is an urgent need to study type 2 diabetic osteoporosis. However, because type 2 diabetic osteoporosis involves many factors, current research on this subject is limited.

Melatonin has many effects on the human body, and has been clinically applied as a pharmaceutical treatment. The function of melatonin in bone has been studied both in vivo and in vitro [3–6]; however, the results have differed according to the concentration administered, so the exact effect of melatonin on bone is not clear [4, 7, 8]. In addition, while it has been reported that different concentrations of melatonin delay osteoblast proliferation [8], few studies have described the effect of melatonin on type 2 diabetic osteoporosis. This study was performed to determine the effects of melatonin in type 2 diabetic osteoporosis, as well as the mechanism of osteoporosis in diabetes mellitus.

Apoptosis and autophagy are involved in multiple physiological and pathological processes. Osteoblast apoptosis has been shown to reduce bone mass, destroy bone microstructure and reduce bone mineral density [9, 10]. Other studies have indicated that autophagy contributes to primary osteoporosis [11]. In previous work, our group identified autophagy as a potential therapeutic target in type 2 diabetic osteoporosis [12]. Although many articles have reported a relationship between melatonin and autophagy, the specific mechanism whereby melatonin regulates autophagy remains controversial [13, 14], and has not been described for type 2 diabetic osteoporosis.

We speculated that melatonin negatively regulates osteoblast autophagy and thus suppresses the pathological process of type 2 diabetic osteoporosis. Therefore, we evaluated the effects of different concentrations of melatonin on in vivo and in vitro models of type 2 diabetic osteoporosis, in order to determine the involvement of melatonin and autophagy in type 2 diabetic osteoporosis and identify potential therapeutic targets for clinical treatment.

## RESULTS

### Model evaluation: in vivo experiments

**Figure 1:**
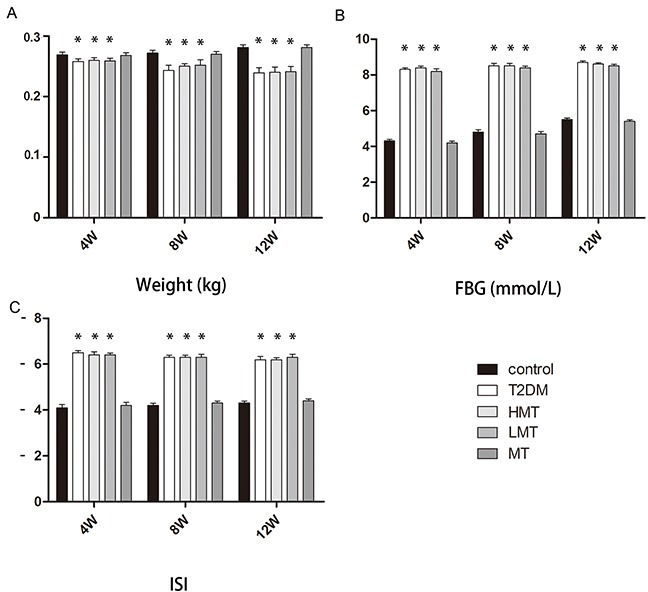
Model evaluation: *in vivo* experiments Forty-five SD rats were used to establish a diabetes model group, and were further divided into the HMT group (n=15, 100 mg/kg melatonin), LMT group (n=15, 50 mg/kg melatonin), and T2DM group (n=15). In addition,15 non-diabetic SD rats were given an intraperitoneal injection of melatonin (75 mg/kg) as the MT group, and 15 non-diabetic SD rats were included in the control group. **A.** Weight analysis indicated that the model animals' weights were lower than those of normal animals at 4,8, and 12 weeks. There was no significant difference between the control and MT groups. **B.** The FBG levels of the model animals were always higher than those of normal animals. There was no significant difference between the control and MT groups. **C.** The ISI levels of the model animals were always lower than those of normal animals. There was no significant difference between the control and MT groups. n=15 per group. Data are means ± SD. *P < 0.05.

In this study, we used a rat model of type 2 diabetes mellitus combined with osteoporosis that was established through the use of intralipids and a small dose of streptozotocin. This widely-used model effectively mimics type 2 diabetes [15–17]. As diabetes is mainly characterized by weight loss, high blood sugar and insulin resistance, we first confirmed that our rat model was a model of diabetes by examining the weights, blood sugar levels, and insulin sensitivity index (ISI) values of the animals. The model animals' weights were significantly lower than those of the normal animals, while their fasting concentrations of blood glucose (FBG)were always higher than those of normal animals. The model animals' ISI values were always lower than those of the normal animals (Figure 1). These results validated our animal model of diabetes.

### Effect of melatonin on bone microstructure

**Figure 2:**
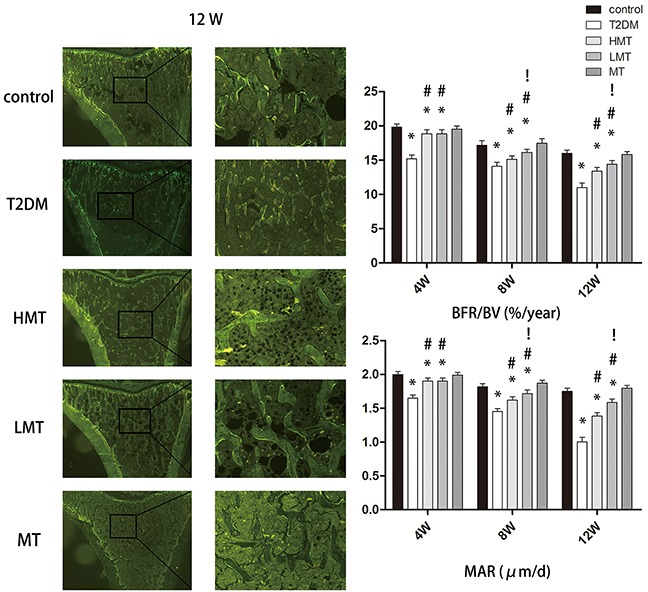
Effect of melatonin on bone microstructure The results of the double-fluorescent labeling method at 12 weeks are shown. The BFR/BV values of the model animals were always lower than those of the normal animals. The BFR/BV values of the LMT and HMT groups were always higher than those of the T2DM group. The BFR/BV values of the LMT group were higher than those of the HMT group at 8 and 12 weeks, although the statistical significance was stronger at 12 weeks. There was no significant difference between the control and MT groups. The MAR values of the model animals were always lower than those of the normal animals. The MAR values of the LMT and HMT groups were always higher than those of the T2DM group. The MAR values of the LMT group were higher than those of the HMT group at 8 and 12 weeks, although the statistical significance was stronger rat 12 weeks. There was no significant difference between the control and MT groups. n=15 per group. Data are means ± SD. *P < 0.05 vs. control, #P<0.05 vs. T2DM group, !P<0.05 vs. HMT group.

**Figure 3:**
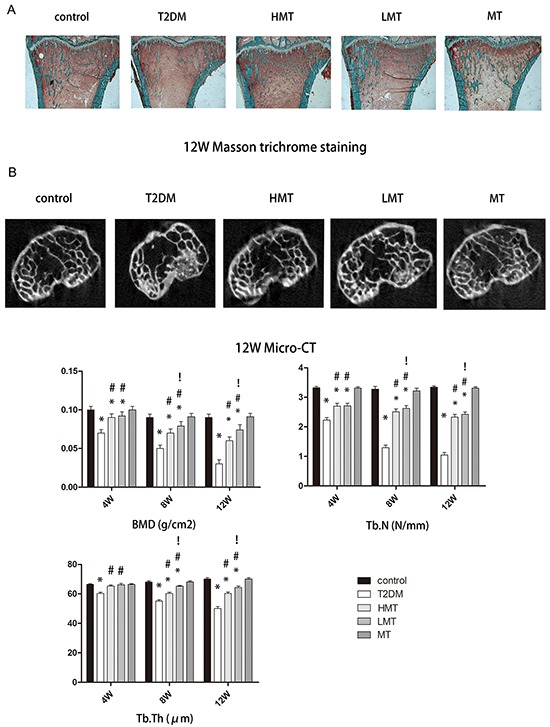
Effect of melatonin on bone microstructure **A.** Masson-Goldnertrichrome staining at 12 weeks. The Tb.Th and Tb.N were significantly lower in the T2DM group than in the control group. Tb.Th and Tb.N were significantly greater in the HMT and LMT groups than in the T2DM group, although greater improvement was seen in the LMT group than in the HMT Group. **B.** Micro-CT scanning at 12 weeks. The BMD values of the LMT and HMT groups were always higher than those of the T2DM group. The BMD values of the LMT group were higher than those of the HMT group at 8 and12 weeks, although the statistical significance was stronger at 12 weeks. There was no significant difference between the control and MT groups. The Tb.N of the LMT and HMT group was always higher than that of the T2DM group. LMT group was always higher than that of the HMT group at 8W, 12W. There was always no significant difference between control group and MT group. The Tb.Th of the LMT and HMT group was always higher than that of the T2DM group. LMT group was always higher than that of the HMT group at 8W, 12W. There was always no significant difference between control group and MT group. n=15 per group. Data are means ± SD. *P < 0.05 vs. control, #P<0.05 vs. T2DM group, !P<0.05 vs. HMT group.

To analyze the effect of melatonin on bone microstructure, we assessed dynamic trabecular bone formation markers including the bone formation rate per unit of bone volume (BFR/BV) and the bone mineral deposition rate (MAR), and static indexes including bone mineral density (BMD), trabecular number (Tb.N), and trabecular thickness (Tb.Th). Based on dynamic and static analysis of the tibia, we observed that the bone structure was significantly worse in the model animals than in the normal animals. We injected additional diabetic rats with a high dose of melatonin (HMT, 100 mg/kg) or a low dose of melatonin (LMT, 50 mg/kg), and measured the above parameters in these rats and in type 2 diabetes mellitus control rats (the T2DM group). The HMT and LMT treatments both promoted the formation of trabecular bone and increased the BMD, Tb.N, and Tb.Th; however, there were greater improvements in the LMT group than in the HMT group. We also compared the same parameters between non-diabetic rats treated with 75 mg/kg melatonin (MT) and non-diabetic controls. No statistically significant differences were detected between the MT group and the control group. which were most pronounced at 12 weeks (Figures 2 and 3). These results suggested that melatonin can improve the bone microstructure of rats with diabetes mellitus.

### Effect of melatonin on bone tissue autophagy

**Figure 4:**
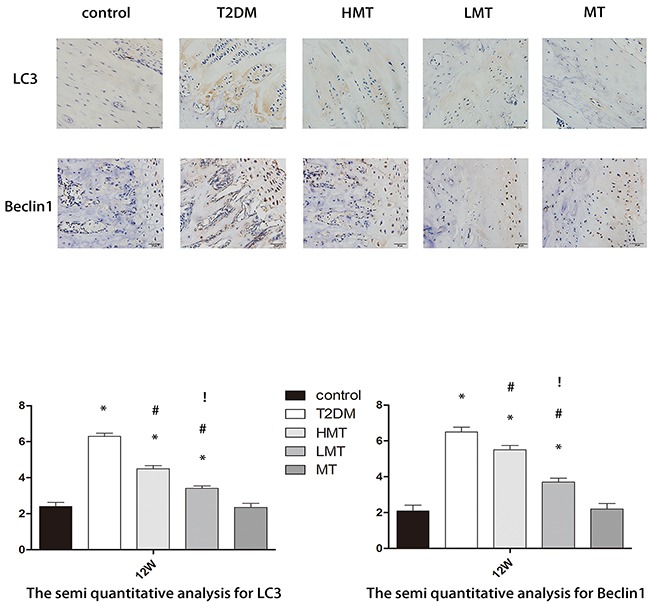
Effect of melatonin on bone tissue autophagy Through IHC staining, we observed the expression of LC3 and Beclin-1 at 12 weeks. LC3 and Beclin-1 protein expression was significantly higher in theT2DM, LMT and HMT groups than in the control group; the expression was lower in the HMT and LMT groups than in the T2DM group, and was lower in the LMT group than in the HMT group. There was no significant difference between the control and MT groups. Scale bars, 20μm. n=15 per group. Data are means ± SD. *P < 0.05 vs. control, #P<0.05 vs. T2DM group, !P<0.05 vs. HMT group.

LC3 and Beclin-1arebiomarkersof the formation of autophagosomes [18]. We used immunohistochemistry (IHC) to detect the levels of these proteins in rat bone tissue. LC3 and Beclin-1werewidely distributed in the cortical bone and can cellous bone. The bone autophagy level was significantly greater in the model animals than in normal animals. However, in the HMT and LMT groups, the levels of autophagy were lower than those of the T2DM group. The difference was more pronounced in the LMT group. There was no statistically significant difference between the MT and control groups (Figure 4). These results suggested that autophagy occurs at a significantly higher level in the bone tissue of rats with diabetes, but that melatonin can reduce the level of autophagy.

### The relationship between high glucose levels and autophagy flux in vitro

**Figure 5:**
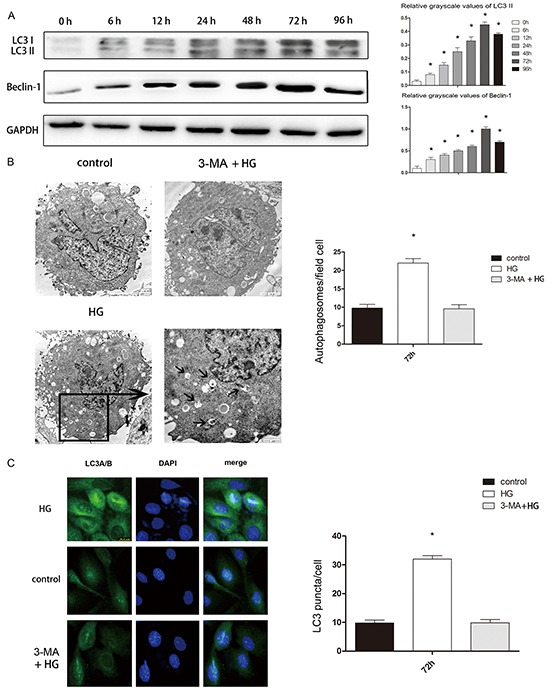
High glucose treatment of hFOB 1.19 cells induced autophagy, which was blocked by 3-MA **A.** Western blot showing Beclin-1 and LC3 protein levels in hFOB 1.19 cells treated with high glucose (HG, 4500mg/L) for 0, 6, 12, 24, 48, 72 or 96 h. n=10 per group. Data are means ± SD. *P < 0.05 vs. 0 h. **B.** TEM results at 72 h. The number of autophagic bodies was significantly greater in the HG group than in the control and 3-MA + HG groups, while there was no significant difference between the control and 3-MA + HG groups. Scale bars, 2μm. n=10 per group. Data are means ± SD. *P < 0.05 vs. control. **C.** IHC results of LC3 at 72 h. The number of LC3 puncta was significantly greater in the HG group than in the control and 3-MA + HG groups, while there was no significant difference between the control and 3-MA + HG groups. Scale bars, 2μm. n=10 per group. Data are means ± SD. *P < 0.05 vs. control.

We next sought to clarify the relationship between autophagy and type 2 diabetic osteoporosis in vitro by using human fetal osteoblastic (hFOB 1.19) cells. To determine whether a high concentration of glucose would promote autophagy in hFOB1.19 cells, we examined the changes in the expression of autophagy-associated proteins including LC3-I/LC3-II and Beclin-1. We found that high glucose levels induced hFOB1.19 cell autophagy in atime-dependent manner, being most significant when the treatment time was 72 h (Figure 5A). Therefore, we used cells treated for72 h for further testing.

We then confirmed that a high glucose concentration induced cell autophagy by counting the number of autolysosomes with a transmission electron microscopy (TEM) and a fluorescence microscope. TEM is recognized as the gold standard for monitoring autophagy. LC3 is mainly located on the surface of pre-autophagosomes and autophagosomes [18]. We used a fluorescence microscope to observe the punctate aggregation of internal LC3 in autolysosomes. We observed the autophagosome quantity by TEM and the LC3 quantity by IHC to determine the level of autophagy. We found that after high glucose treatment, the cell autophagy level increased significantly (Figure 5B, 5C).

**Figure 6:**
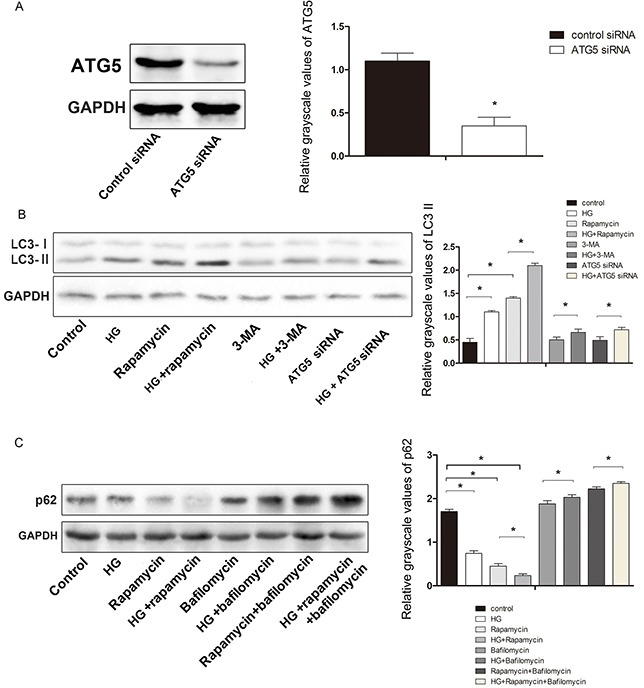
Effect of HG on the expression of the autophagy-related proteinsATG5, LC3, and p62 in hFOB 1.19 cells **A.** Western blot showing ATG5 protein levels in hFOB 1.19 cells. The expression of ATG5 was significantly lower in cells treated with *ATG5* siRNA than in control cells. **B.** Western blot showing LC3-II protein levels in hFOB 1.19 cells treated with HG, rapamycin, *ATG5* siRNA and 3-MA. **C.** Western blot showing p62 protein levels inhFOB1.19 cells treated with HG, rapamycin and bafilomycin. n=10 per group. Data are means ± SD. *P < 0.05.

Macroautophagy can be activated in cells treated with the mTOR inhibitor rapamycin, which mimics metabolic deprivation [19], but autophagic flux inhibition is the suspected mechanism by which bafilomycin A1 promotes autophagosome accumulation [20]. In addition to this, 3-methyladenine (3-MA) has been used asan inhibitor of autophagic sequestration [21]. Inhibition of autolysosome fusion is expected to stabilize LC3-II and p62/SQSTM (p62), an autophagy cargo-identifying protein that is degraded upon autolysosome fusion. Knockdown of ATG5 with small interfering RNA (ATG5 siRNA) inhibited high glucose-induced autophagy flux. The expression of LC3-II was significantly higher in high-glucose-treated osteoblasts than in control cells (not treated with high glucose) (Figure 6A, 6B). To examine the effects of high glucose levels on the autophagic substrate, the levels of p62 were measured in cultures of hFOB1.19 cells treated with high glucose in the presence/absence of bafilomycin A1. High glucose treatment of neuronal cultures in the presence of bafilomycin A1 increased the levels of p62 protein, while high glucose treatment alone reduced the p62 levels (Figure 6C). Treatment with both high glucose and rapamycin further reduced the levels of p62, whereas high glucose treatment along with rapamycin enhanced the levels of p62 in the presence of bafilomycinA1 (Figure 6C). These results suggested that a high glucose concentration induces autophagic flux rather than inhibiting autophagic proteolysis.

### Effects of melatonin on osteoblast autophagy and osteogenic capability

**Figure 7:**
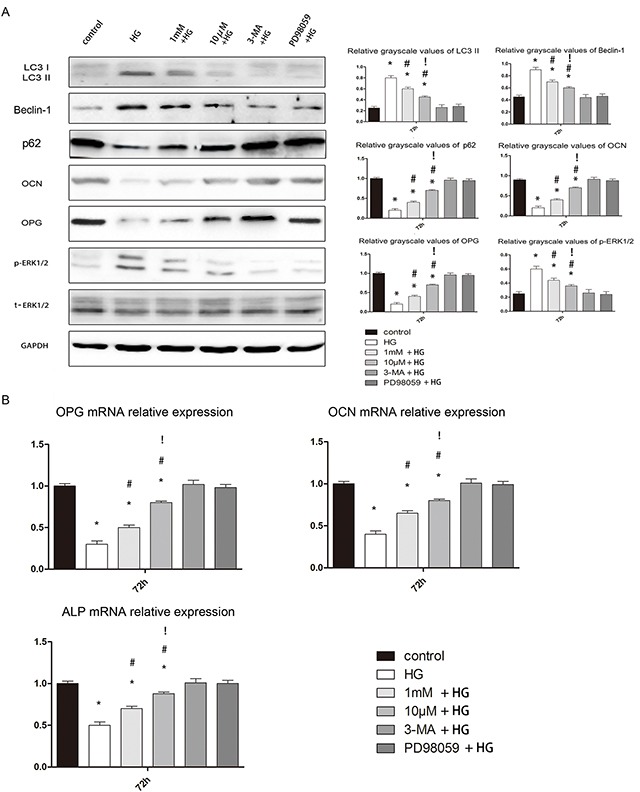
Effects of melatonin on osteoblast autophagy and osteogenesis **A.** Western blotting results at 72 h. The protein expression of LC3-II, Beclin-1 and p-ERK1/2 was always higher in the HG, 1 mM + HG, and 10 μM + HG groups than in the other groups. There were no significant differences between the control, 3-MA + HG and PD98059 + HG groups. The expression of these three proteins was lower in the 1 mM + HG and 10 μM + HG groups than in the HG group, and was lower in the 10 μM + HG group than in the 1 mM + HG group. The protein expression of p62, OCN and OPG was always lower in the HG, 1 mM + HG, and 10 μM + HG groups than in the other groups. There were no significant differences between the control, 3-MA + HG and PD98059 + HG groups. The expression of these three proteins was higher in the1 mM + HG and 10 μM + HG groups than in the HG group, and was higher in the 10 μM + HG group than in the 1 mM + HG group. n=10 per group. Data are means ± SD. *P < 0.05 vs. control, #P<0.05 vs. HG group, !P<0.05 vs. 1mM + HG group. **B.** Real-time PCR results at 72 h. The mRNA expression of *OCN*, *OPG* and *ALP* was always lower in the HG, 1 mM + HG, and 10 μM + HG groups than in the other groups. There were no significant differences between the control, 3-MA + HG and PD98059 + HG groups. The expression of these three genes was higher in the 1 mM + HG and 10 μM + HG groups than in the HG group, and was higher in the10 μM + HG group than in the 1 mM + HG group. n=10 per group. Data are means ± SD. *P < 0.05 vs. control, #P<0.05 vs. HG group.

We next determined the expression of the autophagy proteins LC3, p62 and Beclin-1in untreated hFOB1.19 cells, as well as in cells treated with high glucose, high glucose plus 10 μM melatonin, or high glucose plus 1 mM melatonin. LC3 and Beclin-1 protein expression were significantly greater in cells treated with high glucose than in untreated cells, and were reduced in cells treated with the two concentrations of melatonin, although 10 μM melatonin was more effective. The expression of p62 was opposite to that of LC3 and Beclin-1. The changes in p-ERK1/2 protein expression were consistent with the changes in Beclin-1 and LC3 expression (Figure 7A).

We also measured the osteoblast osteogenic capability in terms of protein and gene expression. Alkaline phosphatase (ALP) and osteocalcin (OCN) promote osteogenesis and are essential for bone mineralization; thus, both can serve as biomarkers of osteogenesis. Osteoprotegerin (OPG) inhibits the function of osteoclasts [22], so reduced expression of OPG can serve as a biomarker of bone resorption. Both concentrations of melatonin significantly enhanced the osteogenic abilities of osteoblasts, although 10μM was more effective (Figure 7B). These results suggested that melatonin may not only suppress autophagy but also enhance osteogenesis in hFOB 1.19 cells treated with high glucose.

## DISCUSSION

We found that melatonin significantly improved bone microstructure and suppressed autophagy in rats with diabetes. In vitro, melatonin both reduced the level of autophagy and improved the osteogenic capacity of osteoblasts.

Melatonin is synthesized from serotonin in the pineal gland after photic information from the retina is transmitted to the pineal gland through the retinohypothalamic tract, suprachiasmatic nuclei, and sympathetic nervous system [23]. Melatonin is closely related to various human pathophysiological processes including iron overload, oxidative stress [24], and diabetes. It has been reported that melatonin can significantly reduce the oxidative stress caused by high blood sugar and lessen the complications of diabetes [25–27]. However, a direct effect of melatonin on blood glucose has not been observed [28, 29]. Our results are consistent with this view.

At present, the involvement of melatonin in osteoporosis remains controversial. Most of the literature suggests that melatonin can improve osteoporosis and promote osteogenesis [30, 31]. However, some studies have noted that melatonin has a negative effect on bone [32]. Based on the existing literature, we chose four melatonin concentrations that respectively have had positive and negative effects in vivo and in vitro:100 mg/kg and 50 mg/kg melatonin for in vivo tests and 10μM and 1 mM melatonin for in vitro tests [7, 8, 33, 34]. Our experiments demonstrated for the first time that melatonin suppresses osteoporosis in diabetes mellitus. Unlike what was previously reported, the effects of melatonin did not differ according to the dose: both high and low concentrations of melatonin improved the bone microstructure and promoted bone formation in vivo and in vitro. As type 2 diabetic osteoporosis is more complex than other types of osteoporosis, so too are its etiology and pathology. Type 2 diabetic osteoporosis involves a variety of factors, including iron overload, oxidative stress, advanced glycation end products (AGEs), etc. [35–37]; this may be the cause of the above phenomenon. Our results indicated the melatonin may be more suitable for the treatment of type 2 diabetic osteoporosis.

We also found that melatonin can suppress autophagy in bone. However, it has been unclear how melatonin regulates autophagy, as previous experiments have shown that the relationship between melatonin and autophagy depends on the environment [13, 14, 38]. We found that both high and low concentrations of melatonin suppressed autophagy in vivo and in vitro. It has been reported that melatonin can inhibit the ERK pathway in osteoblasts [39], and that the ERK pathway promotes the autophagy of osteoblasts [40]. Thus, we speculated that melatonin may suppress autophagy by inhibiting the ERK pathway in osteoblasts. Through disruption of the ERK pathway, we confirmed the regulation of autophagy by melatonin in the osteoblasts of type 2 diabetic osteoporosis.

Our innovative experiment also made clear the involvement of autophagy in type 2 diabetic osteoporosis. Autophagy is a mechanism whereby eukaryotic cells enzymatically degrade intracellular pathogens or damaged organelles to recycle their own [41]. Autophagy participates in the regulation of osteoblasts and osteoclasts and has a close correlation with osteogenesis and bone absorption [42, 43]. We found that each index of bone structure was better in the low-level autophagy group than in the high-level autophagy group, both in vivo and in vitro, confirming the negative effects of autophagy on bone.

The relationship between high glucose levels and autophagy has been studied in a variety of diseases, but has remained unclear. High glucose levels have already been shown to inhibit autophagic turnover in several cell types, such as renal proximal tubular cells and pancreatic cells [44, 45]. However, other studies have indicated that high glucose levels can activate autophagy in cranial neural crest cells and endothelial cells [46, 47]. Research on the relationship between high glucose levels and autophagy in osteoblasts has been limited. High sugar levels were shown to activate autophagy in mouse-derived osteoblasts (MC3T3-E1 cells) [48], and our results in human-derived osteoblasts (hFOB1.19 cells) are consistent with this finding.

Reactive oxygen species (ROS) can stimulate the pathogenesis of diabetic osteoporosis, postmenopausal osteoporosis and glucocorticoid-induced osteoporosis [49–53]. High glucose levels were shown to induce the autophagy of osteoblast cells (MC3T3-E1 cells) by activating the ROS-AKT-mTOR axis [48]. It has been demonstrated in a variety of cell models that ROS induce autophagy by activating the ERK pathway [54–57]. Combining the existing literature with the results of our experiments, we propose that high glucose levels increase of the production of ROS, and that ROS promote autophagy by activating the ERK pathway in osteoblast cells. Melatonin has been confirmed as a classic antioxidant based on its inhibition of ROS production [58]. Therefore, melatonin may reduce osteoblast cell autophagy in diabetic osteoporosisby inhibiting the production of ROS.

The use of laboratory animals has enabled the unprecedented recent improvements in the management of osteoporosis. A variety of methods and animals have been used to create animal models of osteoporosis, each of which has its own advantages, disadvantages and scope of use [59, 60]. Because our research goal was to explore the mechanism of diabetes complicated by osteoporosis, we needed a model that would imitate the pathological process of diabetes. In this study, we used a rat model of type 2 diabetes mellitus combined with osteoporosis that was established through the use of intralipids and a small dose of streptozotocin. This model effectively mimics type 2 diabetes, and is widely used [15–17]. As for the age of the rats, three-month-old rats are sexually mature, six-month-old rats have mature bones, and 17-month-old rats are relatively old [61]. To ensure that we had a successful model of age, we used rats greater than six months old, as their bones were fully mature and thus could undergo diabetic osteoporosis. The bone characteristics of these rats sufficiently reflected the effects of type 2 diabetic osteoporosis.

In conclusion, melatonin can inhibit autophagy, enhance bone microstructure and promote osteoblast osteogenesis by suppressing the ERK pathway in type 2 diabetic osteoporosis.

## MATERIALS AND METHODS

Our experimental design fully complied with the randomized controlled trial principle.

### Ethics statement

The institutional Ethics Review Board of the First Hospital of China Medical University approved the study. The use of animals in our experiments was consistent with ethical requirements. All activities associated with this research project were performed in accordance with the First Hospital of China Medical University Institutional Guidelines and Clinical Regulations.

### Experimental animals

Four-month-old male specific-pathogen-free Sprague Dawley (SD) rats weighing 200±20 g were purchased from China Medical University, Department of Experimental Animals (Animal Certificate Number: SCXK (Liaoning) 2008-0005). To determine the targets of bone histomorphometry, we included a total of 75 SD rats. Forty-five rats were used to establish a diabetic model group, of which15 were given an intraperitoneal injection of melatonin (100 mg/kg) as the HMT group, 15 were given an intraperitoneal injection of melatonin (50 mg/kg) as the LMT group, and 15 were included in the T2DM control group. In addition to these 45 diabetic model rats, 15 non-diabetic SD rats were given an intraperitoneal injection of melatonin (75 mg/kg) as the MT group, and 15 non-diabetic rats were included in the control group.

### Models and specimen collection

The rats received a high-fat diet for two months and were allowed water for 12 hours/day. Establishment of a rat model of type 2diabetes usually involves a substantial injection of streptozotocin (60 mg/kg). However, in order to better simulate the results of diabetes mellitus (relatively reduced insulin secretion), we intraperitoneally injected rats with streptozotocin at a dose of 30 mg/kg. After 72 h, fasting plasma glucose of 7.8 mmol/L and reduced insulin sensitivity were observed, confirming the successful establishment of the model [15]. The rats not used for the model were fed a normal diet. All rats were housed under standard laboratory conditions and maintained under controlled temperature (22 ± 3°C) and humidity conditions with a daily cycle of 12 h light and 12 h dark. The weights of the rats were maintained between 220 g and 270 g, and blood glucose was maintained between 5 mmol/L and 18 mmol/L. Rats with values outside these ranges were eliminated. The rats were killed by cervical dislocation at 4, 8, or 12 weeks after the establishment of the model, and their tibias were immediately aseptically removed, placed into a fresh 4% phosphate-buffered formalin solution and stored at 4°C.

### Bone histomorphometry measurements

All rats were given injections of tetracycline at a dose of 25 mg/kg on the 14th and 13thdays and calcein at a dose of 5 mg/kg on the 4th and 3rd days before they were killed, for the purpose of double labeling in vivo. The right proximal tibial metaphysic was performed on undecalcified sections for bone histomorphometric analysis. BFR/BV and MAR were measured as physical parameters. All bone tissue did not undergo decalcification processing.

We used the above double-fluorescent labeling method along with Masson-Goldner trichrome staining and Micro-CT scanning to measure bone histomorphometry. For Masson-Goldner trichrome staining, slides were deparaffinized and sections were rehydrated, thenmordanted in Bouin's Solution at room temperature overnight undera hood. Slides were washed in running tap water to remove the yellow color from the sections, and stained in working Weigert's iron hematoxylin solution for fiveminutes. Slides were washed in running tap water for five minutes, stainedin Biebrich Scarlet-Acid Fuchsin for five minutes, rinsed in deionized/distilled water, and placed in phosphomolybdic/phosphotungstic acid solution for five to ten minutes. Sections were stained in Aniline Blue Solution for five minutes, rinsed briefly in distilled water, and placed in 1% acetic acid solution for three to five minutes. The slides were dehydratedin xylene overnight to obtain good clearing of the ethanol. Coverslipswere applied with Permount or Polymount.

For Micro-CT scanning, the handle for the right proximal tibial metaphysic (truncated) was fixed along the long axis perpendicular to the specimen in the sample holder. In Viva CT 40, the following scan parameters were selected: image matrix of 1024 × 1024, integration time of 200 ms, energy/intensity of 70 kVp, 114 μA, and 8 W. After the scan was complete, a cancellous bone area (1.0 mm by 3.0 mm in thickness)was selected from the distal growth plate, and the lowest threshold of 190 extracts of image information was used to make a line of reconstruction. After images were recombined through Micro-CT, quantitative analysis was performed with the software. The physical parameters were BMD, Tb.N, and Tb.Th.

### Plasma measurements

Venous blood (tail vein) was collected before experimentation so that the FBG could be measured (Roach blood glucose instrument). Intraocular angular vein blood (2.5-4 mL) was collected for measurement of fasting plasma insulin (FINS) by radioimmunoassay (3v-diagnostic Bioengineer, Shandong, China) and plasma estrogen by ELISA (Rat Oestrogen/E ELISA Kit, 3v-Diagnostic Bioengineer, Shandong, China). The ISI was calculated as the -ln(FINS•FPG) [62].

### Cell culture and materials

The human fetal osteoblastic cell line hFOB 1.19, kindly provided by Dr. M. Subramaniam [63], was maintained in a 1:1 mixture of Ham's F12 Medium and Dulbecco's Modified Eagle Medium without phenol red (Gibco, USA), supplemented with 10% fetal bovine serum (FBS) (HyClone, USA) and 0.3 g/L G418 (Sigma, USA) in a humidified 5% CO2 atmosphere at 33.5°C; the medium was changed every other day. The cells were sub-cultured with trypsin-EDTA and replaced prior to the experiment. The hFOB 1.19 cells were plated at 104 cells/cm2 for 24 h before treatment. The cells were treated with one of the following: high glucose (4500mg/L), the HG group;1 mM melatonin + high glucose, the 1 mM + HG group;10 μM melatonin + high glucose, the 10 μM + HG group; the autophagy inhibitor 3-Methyladenine (3-MA) (5 mM) + high glucose, the 3-MA + HG group; or the ERK pathway inhibitor (PD98059)(50 μM) + high glucose, the PD98059 + HG group. Normal hFOB 1.19 cells were used as the control group.

Melatonin, bovine serum albumin (BSA),3-MA, rapamycin, bafilomycin A1 and PD98059 were obtained from Sigma (St. Louis, MO, USA). Primary antibodies for LC3, p62, t-ERK1/2 (total-ERK1/2), and p-ERK1/2 were purchased from Cell Signaling Technology (CST, USA), while those for Beclin-1, OPG and OCN were purchased from Abcam (USA).

### RNA interference

Lipofectamine 2000 was used to transfect hFOB 1.19 cells with ATG5 siRNA (oligoID HSS114104: Invitrogen, Carlsbad, CA, USA) according to manufacturer's instructions. After a 48-hr culture, knockdown efficiency was measured at the protein level by immunoblot analysis. Nonspecific siRNA (oligoID 12935-300: Invitrogen) was used as the negative control.

### TEM

The cells from each group were digested after 24 h of culture, followed by centrifugation, and the floating cells were collected. The cells were washed twice with cold PBS and fixed in 5% glutaraldehyde. Subsequently, the cells were conventionally dehydrated, embedded, sectioned, and stained, and the formation of autophagosomes was observed by TEM. The number of intracellular autophagosomes in every 10fields was counted.

### IHC

Cells were fixed with 4% paraformaldehyde at room temperature for 15 min. After being washed with PBS, cells were permeabilized with 0.2% Triton X-100 for five minutes. After an additional wash with PBS, sections were incubated in a blocking buffer containing 5% BSA for 30 min at room temperature, followed by incubation with anti-LC3 oranti-Beclin1 antibody overnight at 4°C. Secondary antibodies labeled with fluorescein (1:500, Abcam, USA) were applied for 120 min. Sections were incubated with 0.1% DAPI for five minutes and washed with PBS, and then cover slips were transferred onto the glass slides. Images were captured on a wide-field fluorescent microscope (Olympus, Japan). Semi-quantitative analysis was performed at 200× magnification per visual field (0.145 mm2) for LC3 and Beclin-1 extravasation, with the use of imaging software (ImagePro Plus 6.0; Media Cybernetics, Bethesda, MD, USA). The mean IOD values were analyzed and averaged. The IHC results were analyzed semi-quantitatively on the basis of the positive cell percentage ratio and tinting strength:± was judged as negative, while+ and + + were judged as positive. For the positive cells, 0%was recorded as zero points, ≤25% was recorded as one point, 26-50% was recorded as two points, 51-75% was recorded as three points, and>75% was recorded as four points. For coloring intensity, no color was recorded as zero points, light yellow was recorded as one point, clay bank was recorded as two points, and brown was recorded as three points. Digital summary and statistical analysis.

### LC3 puncta expression analysis

The cells from each group were seeded into 24 wells (25,000 cells/well) and incubated for 12 h. The cells were transiently transfected with the pEGFP-LC3B plasmid with the use of Lipofectamine 2000 (Invitrogen, USA). Briefly, the cells were transfected with 4.0 μg vector DNA and 10 μL Lipofectamine 2000 in 2 mL Opti-MEM medium. Six hours after transfection, the medium was replaced with normal DMEM-F12 medium with 10% FBS for 24 h. Images were captured with wide-field fluorescent microscopy (Olympus, Japan). The number of EGFP-LC3 dots was determined by manual counting in five fields, and the number of nuclei was evaluated through counting of DAPI-stained nuclei in the same fields at the same magnification. The number of LC3 puncta/cell was calculated as the total number of dots divided by the number of nuclei in each microscopic field.

### Western blotting

After treatment, the cells were extracted with lysis buffer (150 mm NaCl, 1% NP-40, 0.1% SDS, 2 μg/mL aprotinin, 1 mm PMSF) for 30 min at 4°C. The supernatants were centrifuged at 12,000 g for 15 min at 4°C. The supernatant containing total protein was harvested. Aliquots containing 50 μg of protein were separated by 12% SDS–PAGE and transferred to PVDF membranes at 60 V or 40 V for two hours at low temperature. The membranes were soaked in blocking buffer (5% skimmed milk) for two hours. Subsequently, proteins were detected with primary antibodies at 1:500 or 1:1000 dilution overnight at 4°C, and were visualized with anti-goat or anti-rabbit IgG conjugated with peroxidase (HRP) at a 1:6000 or 1:8000 dilution, respectively, for two hours at room temperature. The EC3 Imaging System (UVP Inc., Upland, CA, USA) was used to identify the specific bands, and the optical density of each band was measured with Image J software (NIH, Bethesda, MD, USA). The relative content of each protein of interest was calculated as the ratio of its optical density to that of GAPDH in the same sample.

### Real-time PCR

Real-time PCR was performed on an ABI Prism 7900HT Fast System (Applied Biosystems, USA) with SYBR Premix Ex TaqTM II (TaKaRa, China). Amplification was carried out in a total volume of 20 μL for 40cycles after initial denaturation (95°C for 30 s), with the following parameters: 95°C for 5 s and 60°C for 30 s. Primer sequences are listed in Table 1, and β-actin was used as an internal control. The relative mRNA expression was quantified by comparison of the cycle threshold (Ct) values. The experimental data were processed by the 2-ΔΔCt method: ΔΔCt = (Ct target-Ct internal control) experiment group-(Ct target-Ct internal control) normal control group. Each experiment was repeated three times.

**Table 1:** Primer sequences used in real- time PCR experiments

Gene	Primer sequence 5′-3′	Product size (bp)
OPG	F: GCGCTCGTGTTTCTGGACA	226
	R: AGTATAGACACTCGTCACTGGTG	
OCN	F: CACTCCTCGCCCTATTGGC	112
	R: CCCTCCTGCTTGGACACAAAG	
ALP	F: AACATCAGGGACATTGACGTG	159
	R: GTATCTCGGTTTGAAGCTCTTCC	
β-actin	F:GACAGGATGCAGAAGGAGATTACT	142
	R: TGATCCACATCTGCTGGAAGGT	

### Statistical analysis

Two-group comparisons were performed with Student's t-test. Multiple group parameter comparisons were performed with one-way analysis of variance followed by Turkey's post-test. A P value less than 0.05 was considered statistically significant. Statistical analysis was performed with the SPSS statistical package (SPSS, Chicago, IL, USA).
